# Microplastics generated when opening plastic packaging

**DOI:** 10.1038/s41598-020-61146-4

**Published:** 2020-03-19

**Authors:** Zahra Sobhani, Yongjia Lei, Youhong Tang, Liwei Wu, Xian Zhang, Ravi Naidu, Mallavarapu Megharaj, Cheng Fang

**Affiliations:** 1 Global Centre for Environmental Remediation, University of Newcastle, Newcastle, 2308 Australia; 2 State Key Laboratory of Urban Water Resource and Environment, School of Environment, Harbin Institute of Technology, Harbin, 150090 China; 3 Institute for NanoScale Science and Technology, College of Science and Engineering, Flinders University, Adelaide, 5042 Australia; 4 School of Textile Science and Engineering, Tiangong University, Tianjin, 300387 China; 5 Institute of Urban Environment, Chinese Academy of Sciences, Xiamen, 361021 China; 6 Cooperative Research Centre for Contamination Assessment and Remediation of the Environment, University of Newcastle, Newcastle, 2308 Australia

**Keywords:** Environmental monitoring, Environmental impact

## Abstract

Millions of tonnes of plastics have been released into the environment. Although the risk of plastics to humans is not yet resolved, microplastics, in the range of 1 μm - 5 mm, have entered our bodies, originating either from ingestion via the food chain or from inhalation of air. Generally there are two sources of microplastics, either directly from industry, such as cosmetic exfoliants, or indirectly from physical, chemical and biological fragmentation of large (>5 mm) plastic residues. We have found that microplastics can be generated by simple tasks in our daily lives such as by scissoring with scissors, tearing with hands, cutting with knives or twisting manually, to open plastics containers/bags/tapes/caps. These processes can generate about 0.46–250 microplastic/cm. This amount is dependent on the conditions such as stiffness, thickness, anisotropy, the density of plastic materials and the size of microplastics.This finding sends an important warning, that we must be careful when opening plastic packaging, if we are concerned about microplastics and care about reducing microplastics contamination.

## Introduction

Microplastics are plastic fragments, fibres, debris or particles in the range of 1 µm - 5 mm^1^. The distribution and abundance of microplastics in the aquatic system, the terrestrial environment and in the atmosphere have significantly increased in recent decades^2,3^. Plastic accumulation in the natural environment is estimated to be 155–265 million tons by 2060^4,5^, while 13.2% of this weight could be microplastics^6^. The major concern with microplastics relates to their high durability in the natural environment, their strong potential for releasing plastic monomers and additives/chemicals, and their extraordinary vector-capacity for adsorbing/accumulating other environmental pollutants^7^. Although their exact toxicity to humans is not yet resolved, microplastics have been detected in human faeces^8^. Once they enter our bodies, however, either through ingestion or inhalation, microplastics have been reported to potentially cause a localised immune response^7^.

Currently, how microplastics enter our body is not entirely clear. It has been assumed that human exposure to microplastics could be through the food chain (ingestion) or due to air inhalation^7,9^. The evidence to support this assumption is that the presence of microplastics has been confirmed in seafood, honey, sugar, sea salt, tap water and even beer^10–13^. Studies have also addressed the atmospheric fallout of airborne microplastics in urban and suburban sites^14,15^, which could account for 13,731–68,415 microplasticsper annum which ingested during the preparation and consumption of our dinner time alone (such as via household fibres)^9^. By studying the caloric intake of 15% of Americans, Cox, *et al*.^10^ recently estimated the annual exposure as 74,000 to 121,000 microplastics per person via inhalation and ingestion.

Microplastics sources are generally categorised as (i) primary sources of microplastics produced directly in industry^16^ and (ii) secondary microplastics generated indirectly from the fragmentation of larger plastic residues. Karami, *et al*.^17^ called microplastic the chief cross-border contaminant due to their low density and high durability. Once introduced into the environment, the fate and transportation of microplastics can be fuelled through wind advection, stormwater runoff, drainage systems and wastewater^18^. Previous studies suggested that the environmental distribution of microplastics can consequences in food contamination via the processing and packaging of the products, as well as the geospatial location of their sources^19,20^. However, the sources of microplastics are still not fully understood. For example, rather than industry sources (primary one) or as the fragements of industial products (secondary one), do we generate microplastics by ourselves in our daily life?

Here, we investigate the possible generation of microplastics during the opening of plastic packages. That is, microplastics could be generated every day, such as when we open a plastic bag to eat chocolate, cut or tear sealing tape to open a package, twist or open a bottle to drink water, beer, etc. We use quartz crystal microbalance (QCM) in combination with Raman and Fourier-transform infrared spectroscopy (FT-IR) to chemically identify microplastic. In the meantime, we employ scanning electron microscopy (SEM) to physically visualise microplastics for further investigation of their morphology.

## Results

### Mass response

**Figure 1:**
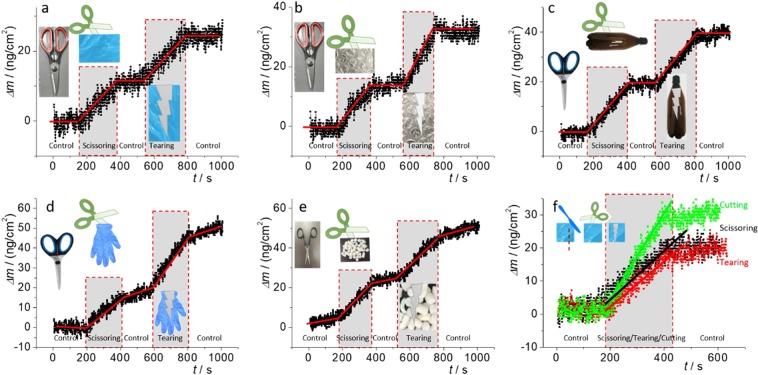
Mass change monitored with QCM during scissoring, tearing and cutting processes, attributed to the generation of microplastics. The insets show the scissors used for scissoring and the plastic targets. “Control” means scissoring air (dry run) before scissoring plastics or when doing nothing before or after scissoring/tearing/cutting plastics. The solid lines indicate the shifting direction of the mass change for clarification and (**f**) compares the different approaches (scissoring, tearing and cutting) for a plastic target.

Figure 1 shows mass changes occurring during scissoring and tearing processes. Different plastic targets were selected and collected from our daily lives, including a shopping bag (a, f), packaging film (b), plastic bottle (c), glove (d) and packaging foam (e). The plastic targets are presented as inserted photos, along with scissors as well. During the 1^st^ “Control” period (1^st^ 200 seconds), we scissored nothing in the air (akin “dry run”). As this study has been done in laboratory conditions, this “Control” was carried out to show the effect of any possible secondary contaminations from surrounding area and from the scissors blades. Then we scissored the selected plastic targets (2^nd^ 200 seconds), followed by doing nothing (3^rd^ 200 seconds, as a “Control” again). We also show a period tearing plastics during the 4^th^ 200 seconds and a further “Control” period (stopping tearing and doing nothing) in the 5^th^ 200 seconds. In general, during the processes of scissoring (200–400 seconds) and tearing (600–800 seconds), a significant change of mass is observed. These mass changes are assigned to the microplastics generated, although the QCM collected only part of microplastics that fell onto its surface. That is, most of the generated microplastics might float in the air or alternatively fall outside the QCM’s working area. Therefore, these results underestimate the mass change and the generated microplastics amount (Supplementary Information).

From Fig. 1, we can see that, regardless of the use of different scissors (here all the scissors were brand-new and supposedly sharp) or the small tip angle at the cutting edge (Supplementary Information, Figs. S1 and S5/6), microplastics generation is possible from various plastic target types, regardless of the scissoring or the tearing approach. The scissoring force leads to deformation and fracture of the target^21^ (Supplementary Information, Fig. S1) and the potential generation of microplastics (modelling in Supplementary Information, Figs. S5/6). Particularly when we consider the gap distance between the two blades (Supplementary Information, Fig. S1b), scissoring acts to slice the plastic target due to shear stress and tension^22^. The microplastics fibres generated by scissoring a bottle or a chocolate bag can be visualised directly by the naked eye (Supplementary Information, Fig. S3, circled). The modelling video also supports this assumption (Supplementary Information, video).

Similarly, during the tearing process, tension is created at the leading point along the tearing direction and potentially leads to breaking of the plastic target into microplastics, particularly when considering the layer structure and the anisotropy of plastic film^23^ (Supplementary Information, Fig. S8). The anisotropy can originate from the manufacture (Supplementary Information, Fig. S2, middle column). Along the tearing boundary, fibres can be visualised again. When zoomed-in, small fibre debris/hairs with diameter of 100–200 nm can be identified, the generation of which depends on the plastic properties again.

Although knife-cutting looks similar to scissoring, the forward-moving driving direction of the former is poorer than that of the latter^21^ (evidenced in Supplementary Information, Fig. S2, right column, Supplementary Information, Fig. S7). The plastic deformation caused by the knife’s cutting edge can also generate microplastic (Supplementary Information, Fig. S2, right column). Needless to say, repeated scissoring/cutting/sliding processes at the same position are akin to sawing to generate mulch.

### Which approach is better?

In Fig. 1(a–e), we can see a 10–70 ng/cm^−2^ mass change. Actually, the amount of microplastics generated depends on many parameters. Here, in order to compare the effects of different opening approaches on microplastics generation, we scissored, tore and cut plastic bags (former supermarket shopping bags in Australia) for the same length (*l*) and under the same forward-moving speed (*v*).

The generation of microplastics shows variable mass changes due to the different approaches to opening the polyethylene (PE) bag. During the opening period, the slope of the line in Fig. 1(f) (in the shadowed area) indicates the mass-changing speed arising from the generation of microplastics.where *ρ*: density; *V*: microplastic size/volume; *N*: number of generated microplastics; *l*: cutting length; *v*: forward speed (*z*-axis here); *H*: stiffness; *J*: fracture toughness; *k*: constant factor related to the elastic properties of the plastic target and the sharpness of the scissors^21^.

Though we cannot control every parameter, we keep the most vital conditions the same for comparisons (in particular, the same *l* & *v*). Thus, a scissoring/tearing/cutting forward-moving length of ~300 cm on a PE bag was tested for 200 sec. In Fig. 1(f), we can see that the mass changes, showing a higher microplastics generation speed/amount for the knife-cutting approach compared to scissoring and tearing, both of which having similar slopes. The modelling results are shown in Supplementary Information.

**Figure 2:**
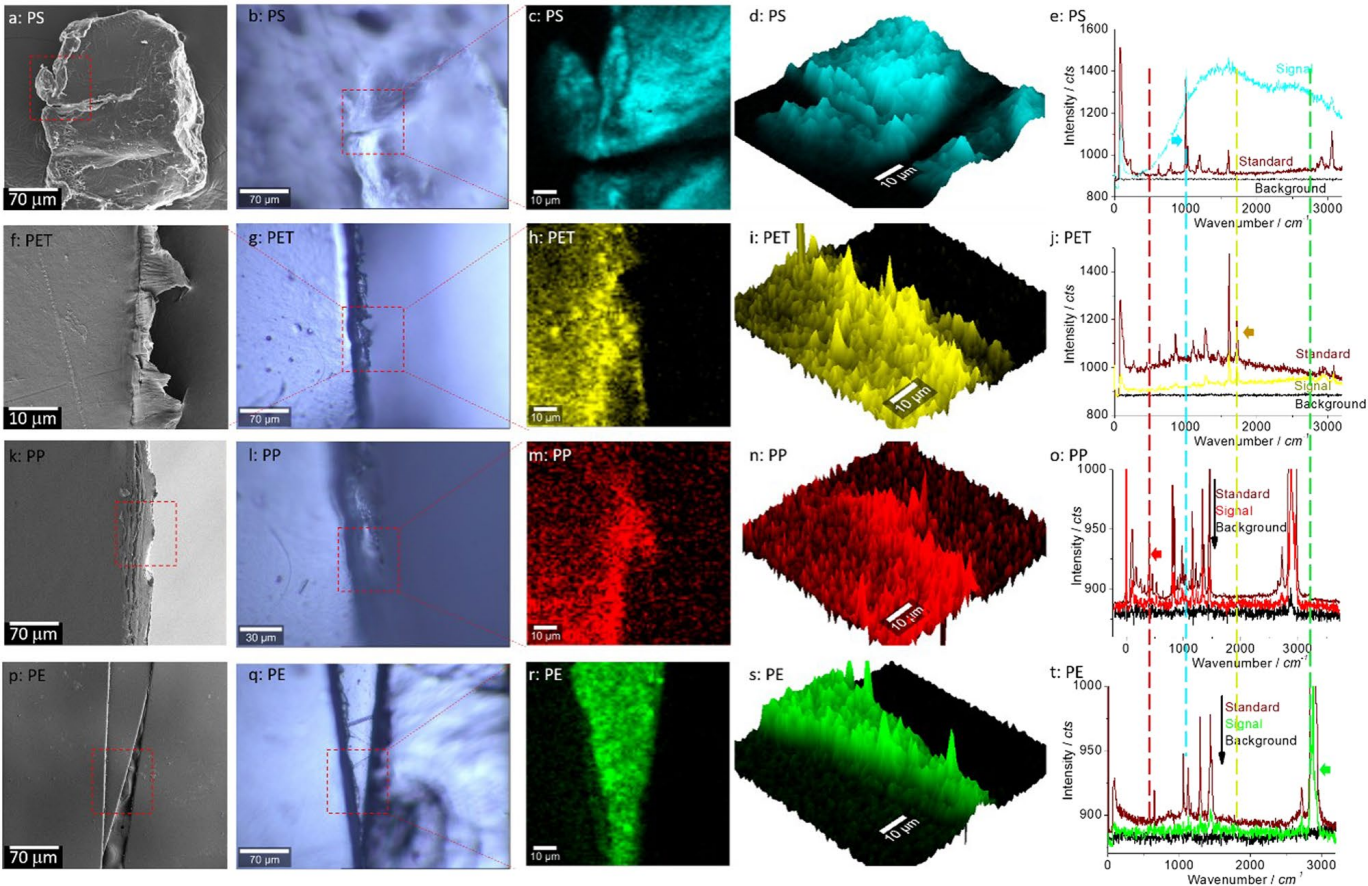
Microplastics confirmed by SEM and Raman spectra. Microplastics particles (**a–e**) are generated by patting packing foam (PS), (**f–j**) by scissoring a drinking-water bottle (PET), (**k–o**) by manually tearing a plastic cup (PP) and (**p–t**) by knife-cutting a plastic bag (PE). From the left column to the right, they are SEM images (showing the boundaries of cutting) (column 1), photo images (column 2), Raman mapping images (columns 3 and 4), and typical Raman spectra (column 5). The laser scanning areas for Raman mapping are suggested by red dashed squares. The characteristic peaks and the typical Raman spectra for Raman mapping and plastic identification are indicated by filled arrows and dashed lines. For comparison, the typical background curve (corresponding to the dark area in the Raman images) and the standard Raman spectra used to identify the type of plastic are also presented.

However, during the scissoring, cutting or tearing processes, different microplastics can be generated randomly in terms of shape (fibre, fragment, triangle, particle, in Fig. 2 and Supplementary Information, Fig. S2) and size (from μm to mm). Moreover, with different plastic materials, aspects of the mass change vary, including stiffness (*H*), thickness (*x*), anisotropy (*y*), the density of plastic materials (*ρ*)^17^. The static electricity of the charged microplastics is another factor that should be considered, because it might lead to a high airborne proportion of microplastics. Perhaps when the charged electrical particlesfall on the QCM surface, interactions arising from static electricity can lead to the phenomenon of reading change/shift during the “Control” periods, as indicated in Fig. 1(e).

### Physical and chemical characterisation

The generation of microplastics during the processes of scissor-scissoring, hand-tearing and knife-cutting, was confirmed by SEM, as indicated in the first column in Fig. 2. To further identify the plastic, chemical characterisation was performed by Raman mapping (3^rd^ and 4^th^ columns of Fig. 2) via microplastics characteristics and fingerprint/signature peaks (right column)^24^. Figure 2(a–e) show an microplastic particle collected by patting a package of polystyrene (PS) foam so that it could originate from debris of manufacture^7^, a scratch on the surface, etc.^25^. Note that this collection is random, and the outcome is unpredictable. This data underpins the importance of washing and cleaning the plastic targets before subsequent testing, as shown in the following text. However, SEM images in Fig. S2 show that some debris might be present even after washing.

The microplastics particle in Fig. 2(f–j) is generated by scissoring a plastic bottle (polyethylene terephthalate (PET)) with a pair of scissors (inset in Fig. 1(a,b)), in Fig. 2(k–o) by hand-tearing a polypropylene (PP) cup, and in Fig. 2(p–t) by cutting a plastic bag (PE) with a knife. The characteristic peaks and the typical Raman spectra for Raman mapping and plastic identification are indicated by filled arrows and dashed lines. Different microplastics, including PS, PET, PP and PE, are visualised and identified by mapping images via their characteristic and fingerprint peaks of 1000 cm^−1^ (for PS), 1720 cm^−1^ (for PET), 402 cm^−1^ (for PP) and 2860 cm^−1^ (for PE), respectively. After Raman mapping, the plastic morphology might have been subjected to a small change from Fig. 2(q) to Fig. 2(p), due to the surface coating (by gold with the thickness of around 30 nm) for the SEM image. Note that Confocal Raman collects its signal mainly from the focal plane. That is the reason why we visualise only the middle “triangle” in the mapping images of Fig. 2(q,r). The left and right parts do not localise in the focal plane (not focused). In the meantime, the microplastics (“daughter” or infant) in Fig. 2(f,k) are still connected to the “mother” plastics but fall down as individual and independent microplastics as shown in Fig. 1 and Supplementary Information, Figs. S5/8 and observed in the video (Supporting Information).

Generally, the SEM images in Fig. 2 showed that most of the generated microplastics shape were fragments and fibers (Supplementary Information, Figs. S2/3 too). This result is in accordance with a previous study by Cox, *et al*.^10^. In Fig. 2(p-s), the two crosswise forward-moving cuts generate a triangular microplastics, because the driving in the forward-moving direction is poor^21^, as also evidenced in Supplementary Information, Fig. S2 (right column).

### Microplastics generation in daily life

**Figure 3:**
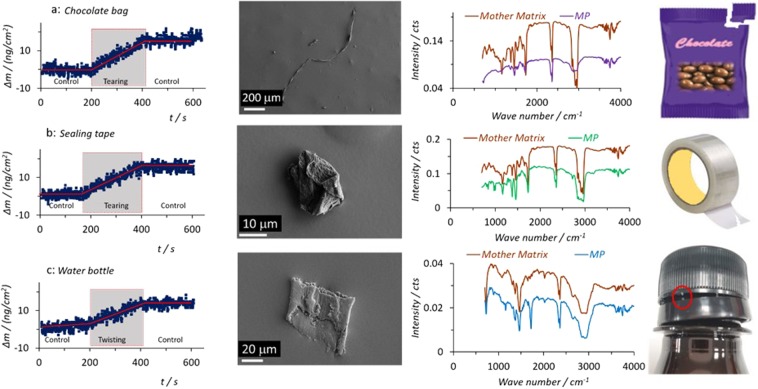
QCM mass changes, SEM images and FTIR spectra of typical microplastics generated in daily life by tearing open a chocolate bag (top row), cutting a sealing tape with a knife (middle row), and twist-opening a plastic bottle (bottom row). FTIR spectra collected from the mother matrices are shown as “Standard” and photo images of the mother matrices are also shown for comparison.

Using the above techniques, the generation of microplastics in our daily lives was monitored during the tearing open of chocolate bags (~300 cm), cutting of sealing tapes (~300 cm), and twist-opening of plastic bottle-caps (~40 bottles), as shown in Fig. 3. The microplastics confirmation was achieved using SEM (to physically show typical/individual microplastics) and FT-IR (to chemically confirm plastic using IR fingerprint/signature spectra, most of them are mainly made of PE)^11^. The fibre in the SEM images in Fig. 3 (top row) is from the tearing of a chocolate bag, particularly when anisotropy of the plastic bag might exist^23^ from the manufacturing, as evidenced in Figs. S2/3. The sealing tape can also generate microplastics (Fig. 3, middle row). However, due to the glue on one side, it is difficult for the microplastics to fall, unless they are self-folded to form a shield on the glue side. The mass changes during twisting-opening the plastic bottle (from a shop-purchased beer container, bottom row) could be due to microplastics generated from a broken junction (cap security ring) or owing to abrasion of the cap by the security ring. Note these plastic materials can vary, depending on the brand/manufacturer of products. In total, the SEM images and FTIR results confirm the generation of microplastics from our everyday activities, even including putting on and taking off clothes when they are made of plastic such as polyester (Supplementary Information, Fig. S4).

## Discussion

Both primary and secondary sources have been described as the microplastics sources in food, water and air^16^. Our work shows that opening plastic packages can generate microplastics in daily life, regardless of the opening approach and plastic target, although the opening approach affects the amount of microplastics generated. These processes can generate a mass change in the range of 10–30 nanograms (Figs. 1/3/S4, via QCM, to collect ~0.05% of the microplastics, discussed below). That is, approximate 14,000–75,000 microplastics (for a 300 cm forward length) are generated, given 0.8–1.4 nanogram per microplastic in mass, based on a cubic dimension of 10 μm × 10 μm × 10 μm. This amount (46–250 microplastic/cm, for a size of 10 μm × 10 μm × 10 μm, or 0.46–2.5 microplastic/cm for a size of 1 mm × 10 μm × 10 μm, Fig. 3) is dependent on the conditions such as stiffness, thickness, anisotropy, the density of plastic materials^17^ and the size of microplastics. For example, as evidenced in Fig. S3, while the big microplastics can be seen by our naked eye, the small ones (Fig. S2) might be also there. With those microplastics generated by ourselves in our daily life as extra sources, we should take our own responsibility when we care about microplastics contaminations.

## Methods

### Materials

Plastic bags, gloves, water bottles, sealing tape and scissors were purchased from local stores in Australia, including Woolworths Supermarkets (plastic bags, scissors), Bunnings Warehouse (gloves, sealing tape), Dan Murphy’s (plastic beer bottle) etc. To remove any contaminants or dust residues or unsealed plastic particles/fibres/debris/residue, all plastic targets, except those in Fig. 2(a–e)), were washed with lab detergent (Decon 90), tap water and Milli-Q water in turn and dried at 40 °C before testing.

### Characterisation

At micro-size, microplastic are estimated to be around 0.8–1.4 nanograms in mass, based on cubic dimensions of 10 μm × 10 μm × 10 μm and given their density (Supplementary Information, Table S1). At this mass level, microplastics cannot be weighed using an analytical balance (with the sensitivity of about 100 micrograms). We, therefore, used a quartz crystal microbalance (QCM) with sensitivity down to 1 nanogram^26^ to monitor the generation of microplastics.

The mass monitoring of the generated microplastic was carried out using a QCM operated via a PGSTAT instrument (Autolab, AUT85423 controlled with Nova 1.1, Netherlands). A 6 MHz gold-coated AT-cut quartz crystal with 0.67 cm^2^ active surface was used as the working area to collect the generated microplastics. Before the experiment, the gold surface was cleaned by washing with a diluted Piranha solution (2: 1 H_2_SO_4_: H_2_O_2_, v/v) (as this solution reacts vigorously with organic compounds, care must be taken while using) to remove potential organic contaminants. This was followed by washing with Milli-Q water and drying by blowing with nitrogen.

The QCM signal was recorded as the resonance frequency shift (∆f). According to the Sauerbrey Eq. (1), (∆f) is proportional to the change in mass (∆m) on the crystal surface^27^.

1 $$\Delta f=-\,{C}_{f}\times \Delta m$$

where *C*_f_ is the sensitivity factor of the crystal which is equal to 0.0815 Hz ng^−1^ cm^−2^ for a 6 MHz AT-cut at 20 °C.

Microplastic were confirmed using a Witec confocal Raman microscope (Alpha 300RS, Germany) equipped with a 532 nm laser diode (<60 mW). A CCD (charged-coupled device) detector (cooled to −60 °C) was used to collect Stokes Raman signals under a 40× objective lens (Nikon, numerical aperture of 0.6) at room temperature over the wavenumber range of 0–4000 cm^−1^ with an integration time of 1.0 s for measurement of a single spectrum or 0.5 s for image mapping. In the latter case, the scanning resolution was fixed at 1 μm/pixel. For more detail see ref. ^25^.

Microplastic were also confirmed using FT-IR conducted on an FT-IR microscope (Agilent, Cary 620 FTIR). The reflection signal was collected under a 15× objective lens (Agilent, numerical aperture of 0.62).

An SEM (Zeiss Sigma VP) equipped with a secondary electron (SE) detector was used to characterise the Microplastic morphology.

### Protocols

The scissors and knife were cleaned with acetone, ethanol and Milli-Q water in turn. Before each test, 10 dry-runs (cutting air) of scissoring air were necessary to avoid any potential metal dust from the cutting edge of the scissor blades. During testing, non-dust gloves were used to avoid interference. All the experiments have been done in laboratory condition.

For plastic scissoring, tearing and cutting, the process was repeated for the length of ~10 cm at the speed of approximately 1.5 cm/s. The cutting/tearing front was moved to retain and align the QCM cell directly and vertically beneath (the vertical distance of around 10 cm) to collect the generated microplastic as they fell. Ideally, QCM is estimated to collect ~0.05% (0.67 cm^2^/4*πr*^2^ with the value of *r* of 10 cm) of the generated microplastic if all the microplastic are generated and distributed uniformly. This assumption is an estimate because most large microplastic is supposed to fall rather than float in the air, due to their higher density than air (Supplementary Information, Table S1).

During the plastic scissoring process, the two blades were positioned symmetrically along with the horizontally-positioned plastic target at the forward-moving speed of ~1.5 cm/s. Far from the scissoring/forward-moving line, the plastic target film might bend a little due to gravity, but the scissoring line was kept horizontal. The length of each scissor cut was 3–10 cm, depending on the length of the scissors’ blade, see Supplementary Information, Fig. S1. That is, once the scissors opened, they were gradually closed, moved forward, and they cut the target to the tip end of the blade. To repeat and continue the scissoring process, the scissors were opened and moved forward horizontally ahead, or to the neighbouring position to cut the target in parallel. This process was repeated 30–100 times within approximately 200 seconds to collect the generated microplastic, with another approximately 200 seconds of scissoring-stopping as a control.

For plastic tearing, plastic targets were scissored to generate a defect/notch/nick array (akin to a sawtooth) in advance and prior to the washing-cleaning process. The plastics were horizontally handled using thumb and forefinger of one hand on one side, and horizontally and symmetrically using thumb and forefinger from the other hand. With two hands’ vertical (up or down) tension, we moved forward to tear the plastic target. Similarly, the QCM was kept vertical under the tearing frontier. The forward-moving direction was not well controlled, compared the situation of scissoring. Therefore, attempts were made to keep the forward movement the same as the scissoring direction. Each tearing distance was also around 10 cm (to tear off and discard the smaller/infant part) and then repeated ~30 times on the larger/mother part at the same moving forward-moving of ~1.5 cm/s. The total torn sample lenght was ~300 cm.

For cutting plastic with a knife, we again held the plastic film horizontally by fixing the four corners of the target with a little tension (not too strong but just enough) to stretch the film and to avoid wrinkling. We pressed the knife down locally to puncture the target film first and then drew/slid the knife to move forward at an angle of ~30° to the plastic film. The cuts were ~10 cm in length and were repeated in parallel. The total cut sample length was ~300 cm.

For twisting a cap, an empty plastic bottle and cap were tested after being washed. The bottle/cap were held horizontally above the QCM. The process was repeated until approximately 40 bottle caps have been opened. Each cap-opening took around 5 seconds at the similar twisting speed.

## Supplementary information

Supplementary information.

Supplementary video.

## References

[1] Hartmann, N. B., Skjolding, L. M., Nolte, T. & Baun, A. In *SETAC Europe 26th Annual Meeting*. 43-44 (SETAC Europe).

[2] Ng E-L (2018). An overview of microplastic and nanoplastic pollution in agroecosystems. Science of The Total Environment.

[3] Jiang J-Q (2018). Occurrence of microplastics and its pollution in the environment: A review. Sustainable Production and Consumption.

[4] Geyer R, Jambeck JR, Law KL (2017). Production, use, and fate of all plastics ever made. Science Advances.

[5] Bergmann M (2019). White and wonderful? Microplastics prevail in snow from the Alps to the Arctic. Science Advances.

[6] Eriksen M (2014). Plastic Pollution in the World’s Oceans: More than 5 Trillion Plastic Pieces Weighing over 250,000 Tons Afloat at Sea. PLoS One.

[7] Wright SL, Kelly FJ (2017). Plastic and Human Health: A Micro Issue?. Environmental Science & Technology.

[8] Schwabl P (2019). Detection of Various Microplastics in Human Stool: A Prospective Case Series. Annals of Internal Medicine.

[9] Catarino AI, Macchia V, Sanderson WG, Thompson RC, Henry TB (2018). Low levels of microplastics (MP) in wild mussels indicate that MP ingestion by humans is minimal compared to exposure via household fibres fallout during a meal. Environmental Pollution.

[10] Cox KD (2019). Human Consumption of Microplastics. Environmental Science & Technology.

[11] Mason, S. A., Welch, V. G. & Neratko, J. Synthetic polymer contamination in bottled water. *Frontiers in Chemistry* **6** (2018). 10.3389/fchem.2018.00407 PMC6141690 30255015

[12] Kosuth M, Mason SA, Wattenberg EV (2018). Anthropogenic contamination of tap water, beer, and sea salt. PLoS One.

[13] Liebezeit G, Liebezeit E (2014). Synthetic particles as contaminants in German beers. Food Additives & Contaminants: Part A.

[14] Klein M, Fischer EK (2019). Microplastic abundance in atmospheric deposition within the Metropolitan area of Hamburg, Germany. Science of The Total Environment.

[15] Dris R (2017). A first overview of textile fibers, including microplastics, in indoor and outdoor environments. Environmental Pollution.

[16] Gregory MR (2009). Environmental implications of plastic debris in marine settings–entanglement, ingestion, smothering, hangers-on, hitch-hiking and alien invasions. Philosophical transactions of the Royal Society of London. Series B, Biological sciences.

[17] Karami A (2017). The presence of microplastics in commercial salts from different countries. Scientific Reports.

[18] Eriksen M (2013). Microplastic pollution in the surface waters of the Laurentian Great Lakes. Marine Pollution Bulletin.

[19] Kim J-S, Lee H-J, Kim S-K, Kim H-J (2018). Global Pattern of Microplastics (MPs) in Commercial Food-Grade Salts: Sea Salt as an Indicator of Seawater MP Pollution. Environmental Science & Technology.

[20] Lee H, Kunz A, Shim WJ, Walther BA (2019). Microplastic contamination of table salts from Taiwan, including a global review. Scientific Reports.

[21] Mahvash M (2008). Modeling the Forces of Cutting With Scissors. IEEE Transactions on Biomedical Engineering.

[22] Burdett C, Theakston M, Dunning J, Goodwin A, Kendall SWH (2016). Left-handed surgical instruments – a guide for cardiac surgeons. Journal of Cardiothoracic Surgery.

[23] O’Keefe R (1994). Modeling the tearing of paper. American Journal of Physics.

[24] Sobhani Z, Al Amin M, Naidu R, Megharaj M, Fang C (2019). Identification and visualisation of microplastics by Raman mapping. Analytica Chimica Acta.

[25] Oßmann BE (2018). Small-sized microplastics and pigmented particles in bottled mineral water. Water Research.

[26] Arnau A, Montagut Y, García JV, Jiménez Y (2009). A different point of view on the sensitivity of quartz crystal microbalance sensors. Measurement Science and Technology.

[27] Tsai W-Y, Taberna P-L, Simon P (2014). Electrochemical Quartz Crystal Microbalance (EQCM) Study of Ion Dynamics in Nanoporous Carbons. Journal of the American Chemical Society.

