# Supplementary material for: Microplastics generated when opening plastic packaging
Source: Sci Rep. 2020 Mar 19;10:4841. doi: 10.1038/s41598-020-61146-4 (PMC7082338; doi:10.1038/s41598-020-61146-4)
Supplement: Supplementary file 1 — Supplementary information. [file 41598_2020_61146_MOESM1_ESM.docx]

# Supplementery information

# Microplastics generated when opening plastic packaging

Zahra Sobhani ^1^, Yongjia Lei ^1, 2^, Youhong Tang ^3^, Liwei Wu ^3, 4^, Xian Zhang ^5^, Ravi Naidu ^1, 6^, Mallavarapu Megharaj ^1, 6^, Cheng Fang ^1, 6, *^

^1^ Global Centre for Environmental Remediation, University of Newcastle, NSW 2308, Australia

^2^ State Key Laboratory of Urban Water Resource and Environment, School of Environment, Harbin Institute of Technology, Harbin 150090, China

^3^ Institute for NanoScale Science and Technology, College of Science and Engineering, Flinders University, South Australia 5042, Australia

^4^ School of Textile Science and Engineering, Tiangong University, Tianjin 300387, China

^5^ Institute of Urban Environment, Chinese Academy of Sciences, Xiamen 361021, China.

^6^ Cooperative Research Centre for Contamination Assessment and Remediation of the Environment, University of Newcastle, NSW 2308, Australia

# Experiments and image gallery

Table S1. The density of general plastic ^1^

| **Plastic type** | PE | PP | PS | PVC | PET | Nitrile rubber |
| --- | --- | --- | --- | --- | --- | --- |
| **Specific density (g•cm^−3^)** | 0.91–0.94 | 0.83–0.85 | 1.05 | 1.38 | 1.37 | 1.28 |


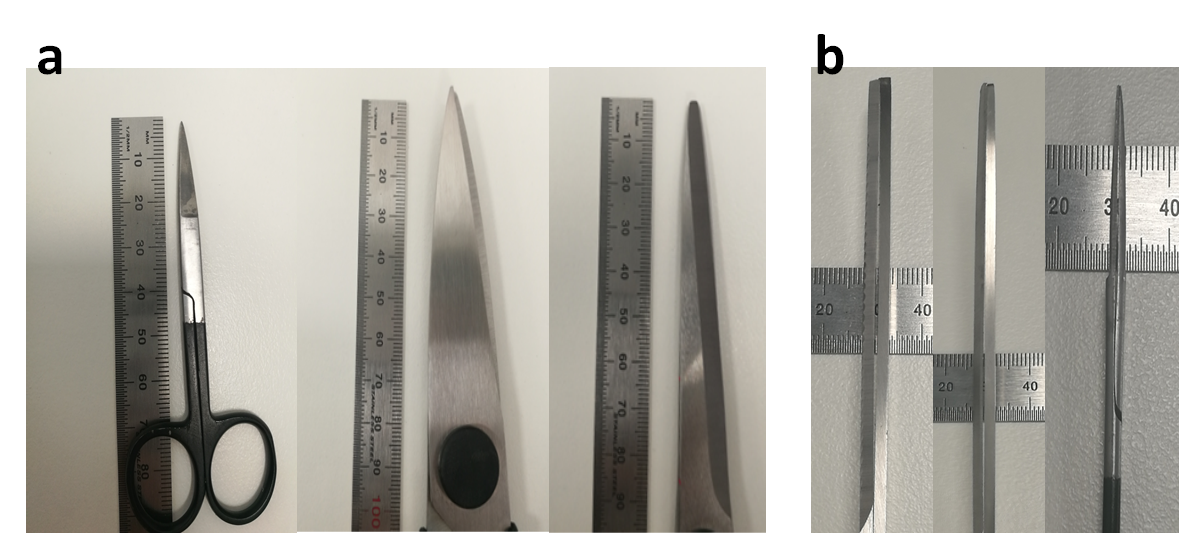


Figure S1: Photo images of scissors, showing (a) length of blades and (b) gap between two blades.


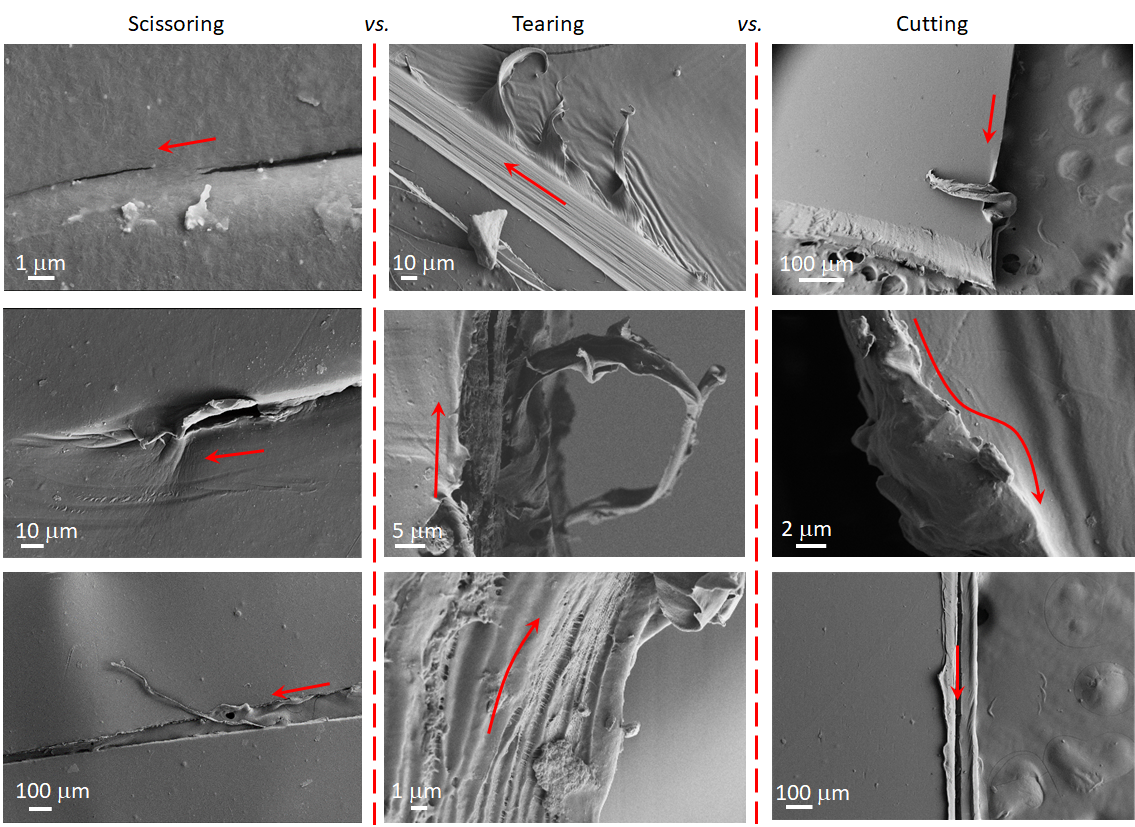


Figure S2: SEM images showing the microplastics (at the early stage, or embryo) generated during scissoring (left column), tearing (middle column) and cutting processes (right column). For the PE-scissoring process (the left column), the top two images show the deformation and fracture at the scissoring frontier, and a generated fibre (the bottom image). For the PE-tearing process (middle column), the layered structure of the plastic film (perhaps dependent on the manufacturing process) can be seen, and the fibres and debris are clearly identified. For the PP-cutting process (the right column), the cutting is drawn and moved forward, and fibre can be observed either along the straightforward cutting line (top), or debris at the change in cutting direction (middle). The deformation of the plastic target is shown in the bottom image. Forward-moving directions are indicated by red arrows.


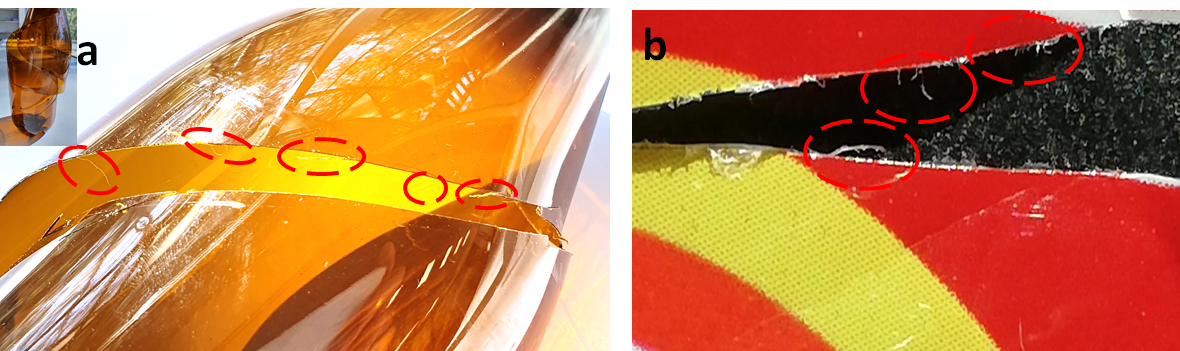


Figure S3: Plastic fibres (circled) generated by scissoring (a) a water bottle and (b) a chocolate bag. Photos by Samsung Galaxy S10 at different magnifications.


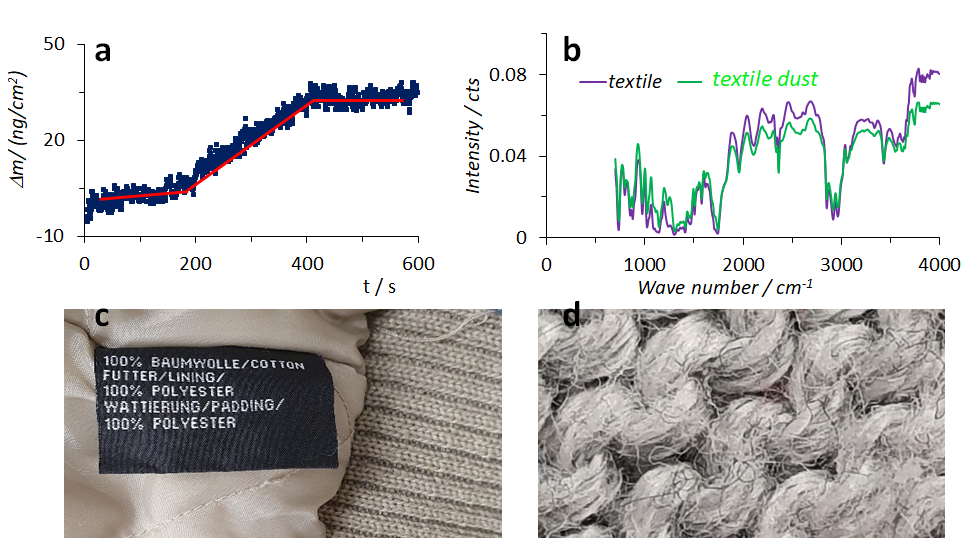


Figure S4: Mass change monitored with QCM (a) typical FTIR spectra (b) for abrasion of polyester textile. (c-d) photo of a jacket (having been washed 5 times in a washing machine) brand tag and the zoomed-in textile. Photos by Samsung Galaxy S10 at different magnifications. For clothing abrasion, a 6-month-old polyester jacket was selected. Before testing, it has been washed 5 times in a washing machine and dried. During testing, small portions of the sleeve fabric were rubbed (as in handwashing) above the QCM cell. The rest was the same as in the other tests.

# 2. Modelling

The dynamic explicit solver of the finite element software ABAQUS was used to conduct the finite element simulation/modelling of the Microplastic generation during scissoring, cutting and tearing processes. PET (polyethylene terephthalate) was selected as an isotropic elastoplastic material in the finite element method (FEM) for modelling. The density of PET is 1335 kg/m^3^ and the elastic modulus is 1345.39 MPa, respectively. Other properties of the selected plastic are shown in Table S2.

Table S2. Plastic properties of PET.

| Yield Stress (MPa) | Plastic properties |
| --- | --- |
| 75.49 | 0 |
| 155.26 | 0.928 |

In Figures S5/7, the modelling focus on the cross-section only, *x-y* axis (0.5 mm × 3 mm), at a selected point, at the moving forward direction that is along the *z*-axis. However, in Figures S2-4, the modelling is a top view of *x-y* plane.

## 2.1. Scissoring


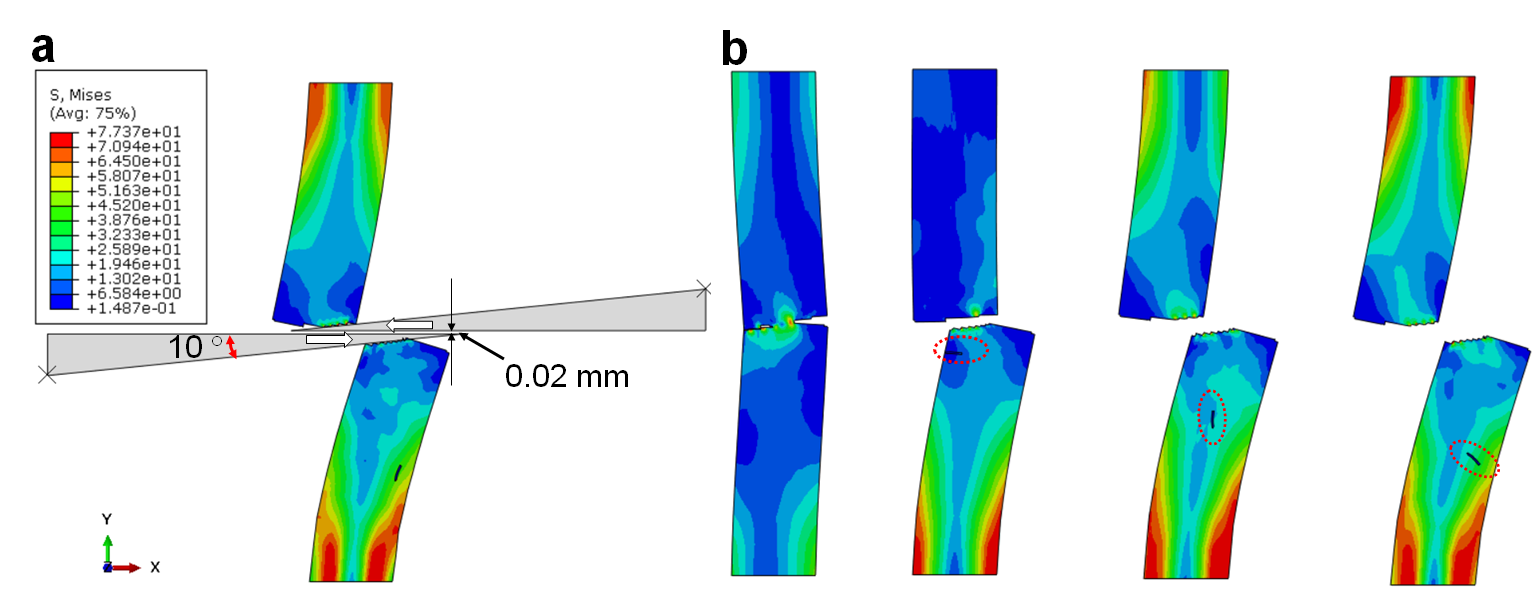


Figure S5: Modelling of the scissoring process. In (a), the gap distance between the two blades (triangles) is 0.02 mm and the tip angle (sharpness) of the blade at the cutting edge is 10°, as indicated. The two white arrows mark the cutting direction of the blades along the *x*-axis. The deformation occurring during the scissoring process is shown in (b) (from left to right, at different scissoring stages). The debris generated is red-circled.

Figures S5 and S6 show the results of FEM of the scissoring process, for which a video is provided in Supplementry Information, video. The sample of the plastic film 0.5 mm × 3 mm (cross-section). For modelling, the scissors have a rigid body with three different tip angles (10°, 20° and 40°) at the cutting edge of the blade, and with four different gap distances (0.02, 0.1, 0.2 and 0.4 mm) between the two blades. The solid unit of CPS4R is used in the model, and the elements of the contact area are condensed. During the scissoring process, shear damage is chosen. The updated damage variables were obtained when the criteria of damage initiation were reached. Subsequently, the stiffness degradation was computed to reveal the shear stress increment of integration points.

In Figure S5, microplastic scissoring debris is visible, falling like a fibre (red-circled). In Figure S6 (a) a larger gap appears between the two blades, and (b) a greater tip angle at the cutting edge generates a larger sized microplastic. In the meantime, the generated debris (daughter or infant) might still stick to the “mother” film (such as marked for 0.2 mm and 20°, respectively), as observed in Figure 2(f) and Figure S1.


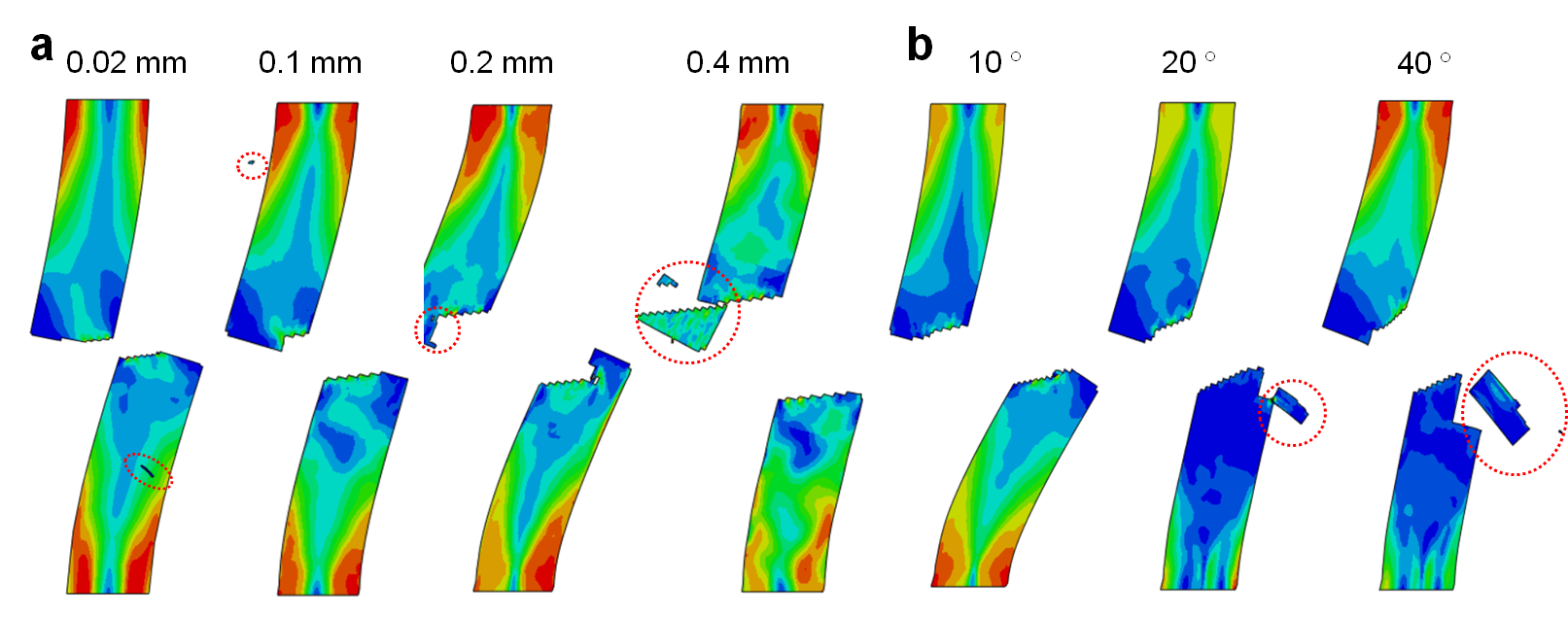


Figure S6: Modelling of the scissoring results, depending on (a) the gap distance between the two blades and (b) the tip angle of the blade at the cutting edge. In (a), the tip angle is fixed at 10° and in (b) the gap distance is fixed at 0.1 mm. For more detail see Figure S2-1.

## 2.2. Knife cutting

At first glance, cutting looks similar to scissoring. However, there is only one blade and no shear stress with knife cutting. Figure S7 shows the FEM of the knife-cutting process. The knife has a rigid body with three different tip angles (10°, 20°and 40°) at the cutting edge for modelling. The other parameters are consistent with the scissors model.


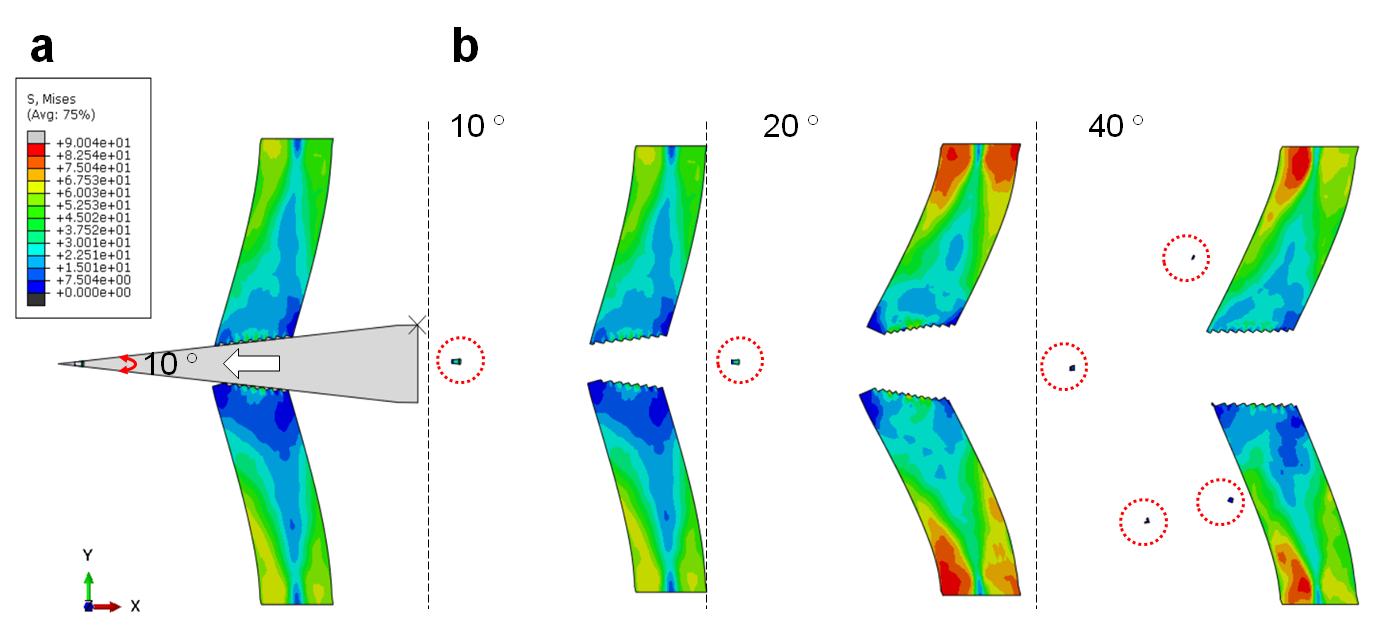


Figure S7: Modelling of the knife-cutting process. The tip angle of the knife at the cutting edge is 10° in (a) and its effect is shown in (b) (from left to right). For more detail see Figure S2-1.

Similar to the scissoring process, a greater tip angle at the cutting edge can generate more microplastic, like a blunt knife cutting. A blunt knife might push and slice a slide of plastic, which is not modelled here. However, this situation could occur and is evidenced in Figure S1 (the top-right image).

The knife seems to punch the plastic film in Figure S7(a). The reason is that the modelling focuses on a selected point of the cross-section. The forward movement of the cutting edge of the knife is along the z-axis. In our case, at the selected cutting point, the knife pushes down (*x*-axis) and splits the plastic film, which is modelled here.

## 2.3. Hand-tearing

The final part is the tearing process of PET, as shown in Figure S8. Basically, the extended FEM (XFEM) is used to describe the nonlinear fracture of PET during the tearing process.

With the anisotropic plastic film, the forward-moving direction could change during the tearing process, as observed in Figure S1 (the middle-bottom image). After tearing, some parts of the plastic are missing (circled red in Figure S8(g)), which are assigned to the fallen microplastic.


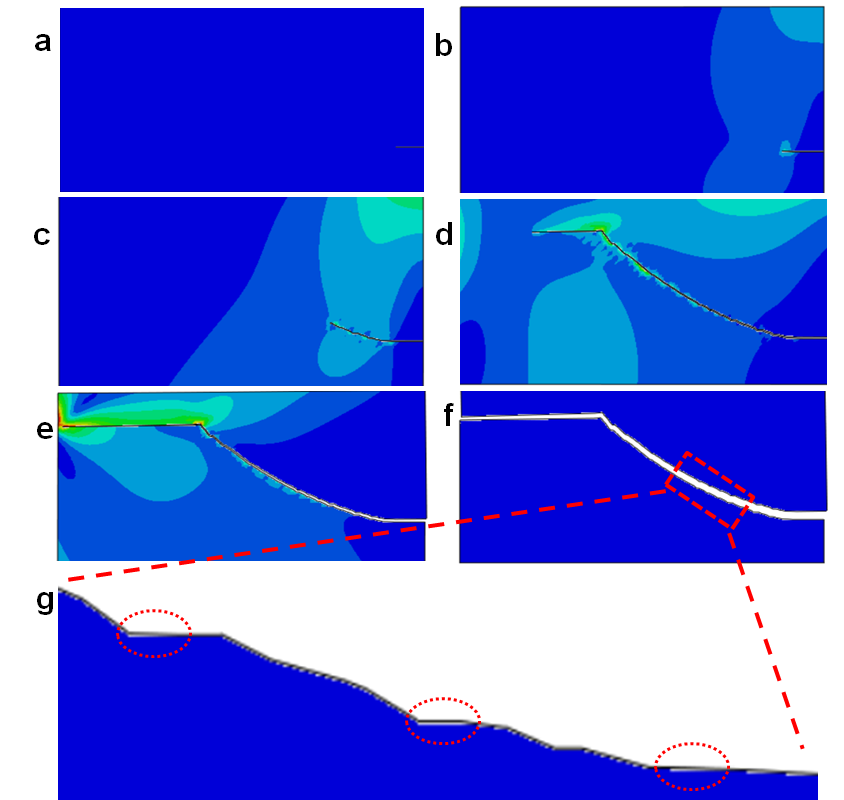


Figure S8: Modelling of the tearing process, from (a) to (f). (g) is the zoomed-in part of (f), showing where parts (as microplastic) of the plastic “daughter” are missing from “mother” after tearing. For more detail see Figure S5.

# References

1 Andrady, A. L. in *Marine Anthropogenic Litter* (eds Melanie Bergmann, Lars Gutow, & Michael Klages) 57-72 (Springer International Publishing, 2015).
